# Importance of underlying mechanisms for interpreting relative risk of *Clostridioides difficile* infection among antibiotic-exposed patients in healthcare facilities

**DOI:** 10.1371/journal.pone.0306622

**Published:** 2024-08-08

**Authors:** Christopher Mitchell, Lindsay T. Keegan, Thuy T. T. Le, Karim Khader, Alexander Beams, Matthew H. Samore, Damon J. A. Toth

**Affiliations:** 1 Department of Mathematics, Tarleton State University, Stephenville, Texas, United States of America; 2 Division of Epidemiology, Department of Internal Medicine, University of Utah, Salt Lake City, Utah, United States of America; 3 Department of Veterans Affairs Salt Lake City Health Care System, Salt Lake City, Utah, United States of America; 4 Department of Health Management and Policy, School of Public Health, University of Michigan, Ann Arbor, Michigan, United States of America; 5 Department of Mathematics, Simon Fraser University, Burnaby, British Columbia, Canada; University of California San Francisco, UNITED STATES OF AMERICA

## Abstract

*Clostridioides difficile* infection (CDI) is a significant public health threat, associated with antibiotic-induced disruption of the normally protective gastrointestinal microbiota. CDI is thought to occur in two stages: acquisition of asymptomatic colonization from ingesting *C. difficile* bacteria followed by progression to symptomatic CDI caused by toxins produced during *C. difficile* overgrowth. The degree to which disruptive antibiotic exposure increases susceptibility at each stage is uncertain, which might contribute to divergent published projections of the impact of hospital antibiotic stewardship interventions on CDI. Here, we model *C. difficile* transmission and CDI among hospital inpatients, including exposure to high-CDI-risk antibiotics and their effects on each stage of CDI epidemiology. We derive the mathematical relationship, using a deterministic model, between those parameters and observed equilibrium levels of colonization, CDI, and risk ratio of CDI among certain antibiotic-exposed patients relative to patients with no recent antibiotic exposure. We then quantify the sensitivity of projected antibiotic stewardship intervention impacts to alternate assumptions. We find that two key parameters, the antibiotic effects on susceptibility to colonization and to CDI progression, are not identifiable given the data frequently available. Furthermore, the effects of antibiotic stewardship interventions are sensitive to their assumed values. Thus, discrepancies between different projections of antibiotic stewardship interventions may be largely due to model assumptions. Data supporting improved quantification of mechanistic antibiotic effects on CDI epidemiology are needed to understand stewardship effects better.

## 1 Introduction

*Clostridioides difficile* (*C. difficile*) is the leading cause of healthcare-associated diarrhea worldwide [1–4]. *C. difficile* infection (CDI) is characterized by diarrhea that can lead to life-threatening colon inflammation and is one of only five pathogens classified by the Centers for Disease Control and Prevention as an urgent threat [5–8]. Each year there are approximately 225,000 CDI hospitalizations and over 12,000 deaths in the United States, and nosocomial CDI more than quadruples the cost of hospitalization [9]. While *C. difficile* can coexist with other gastrointestinal organisms, its growth is normally suppressed by more dominant anaerobic bacteria [10]. Antibiotic exposure can increase susceptibility to *C. difficile* colonization and progression by depleting the normal intestinal microbiota.

Certain classes of antibiotics have been shown to put patients at increased risk for *C. difficile* [4, 11, 12]. The relationship between antibiotic exposure and CDI indicates an opportunity to reduce the burden of CDI through antibiotic stewardship, especially given that up to 30% of all antibiotic prescribing in the United States is considered unnecessary or inappropriate [11]. Although previous studies have shown that hospital patients with recent exposure to certain classes of antibiotics are more likely to develop CDI than patients without exposure [11, 13], the relationship between antibiotic exposure and either acquisition of *C. difficile* or progression from colonization to CDI is not well understood.

Multiple previous studies have used dynamical models to explore the role of antibiotics on CDI [14–21]. These papers make different assumptions about the effect of antibiotics on the acquisition of *C. difficile*, the effect of antibiotics on the progression to CDI, and the resulting effectiveness of antibiotic interventions. Some studies assume that antibiotics affect both acquisition and progression and vary in their findings on the strength of interventions [14, 16, 17]; other studies assume that antibiotics only affect progression to CDI and again vary in their findings on the strength of interventions [15, 19, 20]; and yet others assume that antibiotics only affect the acquisition of *C. difficile* and conclude that the effect of interventions is strong [9, 18]. Models assuming that antibiotics affect CDI progression but not acquisition consistently produce lower estimates for intervention effectiveness than their counterparts which assume otherwise, suggesting that a priori assumptions built into models may be constraining their predictive capability.

The lack of consensus in the literature on how antibiotics affect *C. difficile* acquisition and progression and the varying magnitude of these effects demonstrates the problem at hand: how do the assumed affects of antibiotics on acquisition and/or the progression to CDI change the outlooks of interventions? Here, to quantify the impact of antibiotics on both acquisition and progression we develop a model of hospital-associated *C. difficile* infections. This was done using a deterministic system of ordinary differential equations. We then apply our model to different classes of antibiotics to understand how these different classes of antibiotics impact susceptibility and progression to understand how this may impact potential interventions including the impact of reducing the frequency of antibiotic prescribing, shortening the duration of high-risk antibiotic courses, and shifting antibiotic prescriptions away from classes linked to CDI.

## 2 Methods

### 2.1 Antibiotic risk state model

Studies that have quantified the degree of increased risk of CDI for certain antibiotic-exposed patients do so by comparing CDI incidence among patients recently exposed to a given antibiotic, antibiotic class, or group of antibiotic classes versus a control group of patients without such recent exposure. While several different antibiotics have been associated with increased CDI risk via results from that type of study, we chose to calibrate our model to results from a particular study producing results for patients exposed to a group of broad spectrum agents that included fluoroquinolones, 3rd and 4th generation cephalosporins, and *β*-lactam/*β*-lactamase inhibitor combinations (the latter group we hereafter refer to as “penicillin combinations” for brevity) [11]. Antibiotics in these classes disrupt the patient’s microbiota leading to an increased risk of CDI, therefore we refer to these classes of antibiotics as “disruptive.” There is some suggestion that carbapenems might be considered disruptive as well as inhibitory. While [11] did not inlcude patients exposed to carbapenems in the anyalsis, other studies [22–26] have found evidence of increased CDI risk with that drug class. For the purpose of the main project we consider only the case described above. We did perform an additional sensitivity analysis in which carbapenems were added to the disruptive group. See S4 Table in S1 File as well as S1-S4 Figs in S1 File for further results.

We also chose to use our model to explore potential nuances related to the timing of entering a CDI-associated risk state during a patient’s hospital stay. Relative risk studies generally do not distinguish between patients in the risk group who experienced CDI onset during the antibiotic course and patients whose CDI onset occurred in a time window after the course was completed, However, there may be a difference in the CDI risk of those groups if the antibiotic course included a drug or drugs with activity against C. difficile in addition to the disruptive effect. Those competing effects could occur if a disruptive antibiotic is taken in combination with a drug used for CDI treatment, such as oral vancomycin or metronidazole [27], or with a broad spectrum drug that have activity against C. difficile, such as carbapenems, penicillins, and penicillin combinations [28–31]. We refer to those five classes of antibiotics as “inhibitory.” We tested a scenario where carbapenems were “not” included in the inhibitory group. See the last row of S4 Table in S1 File.

Because we placed penicillin combinations in both the disruptive and inhibitory groups, the assumed effect on patients of receiving a drug in this category is that they enter an elevated CDI risk state only at the completion of the antibiotic course, when the inhibitory effect is no longer active. By contrast, patients receiving the other drugs in the disruptive group (fluoroquinolones and 3rd and 4th generation cephalosporins) with no other inhibitory drugs in combination are assumed to enter the elevated risk state at the start of the antibiotic course. Our assumption for delaying the timing of elevated CDI risk for patients receiving penicillin combinations is supported by a study finding that piperacillin/tazobactam achieved concentrations in the intestinal tract during therapy that were high enough to inhibit C. difficile colonization [28]. For more on these assumed antibiotic categories see Section 2.3.

For simplicity we assumed only two levels of patient risk states with respect to C. difficile acquisition and/or infection: elevated and normal. After admission, patients can transition to the elevated state *V* from either state *N* (by beginning a new antibiotic course) or from state *A* (by ending an antibiotic course). The transition to the elevated risk state can be entered either at the beginning of an antibiotic course consisting of only disruptive antibiotics, or at the completion of an antibiotic course containing both disruptive and inhibitory antibiotics, as described above. All patients not in the elevated risk state are assumed to be at the same, lower level of C. difficile risk regardless of antibiotic exposure. Thus, the only assumed effect of inhibitory antibiotics is to cancel out the would-be effect of a disruptive antibiotic.

Patients with normal C. difficile risk are in state *N* if not currently on antibiotics and state *A* if currently on antibiotics. Patients with elevated risk are in state *V*, whether they are currently on antibiotics or not. For simplicity, we assume that patients entering an elevated risk state remain elevated until the end of the hospital stay regardless of remaining stay duration.

The antibiotic risk state model is:

$$\begin{array}{c} \begin{array}{cc} \frac{dN}{dt} & =1-\left(f_{A}+\omega_{0}\right)+\left(1-\sigma \right)\mu A-\left(\omega +1\right)N\\ \frac{dA}{dt} & =\left(1-\lambda \right)\left(f_{A}+\omega_{0}\right)+\left(1-\lambda \right)\omega N-\left(\mu +1\right)A\\ \frac{dV}{dt} & =\lambda (f_{A}+\omega_{0})+\lambda \omega N+\sigma \mu A-V \end{array} \end{array}$$ (1)

The state *N* represents normal-risk patients on no antibiotics, *A* represents normal-risk patients on antibiotics, and *V* represents all elevated risk patients. The parameter *f*_*A*_ is the probability of newly admitted patients already on antibiotics; *ω* is the rate of starting a new antibiotic course during the stay (and *ω*_0_ is the rate of starting a new antibiotic on admission); and *μ* is the rate of stopping an antibiotic course during the stay. Those four parameters are based on starting and stopping rates of any antibiotic administration to hospital inpatients, regardless of antibiotic type. Finally, the parameters *ω* and λ govern the rates of transition into the elevated C. difficile risk state *V*. The parameter λ is the probability that a patient starting an antibiotic course enters the elevated risk state at the same moment, i.e. the fraction of all antibiotic courses that contain a disruptive antibiotic and no inhibitory antibiotic. Antibiotic courses that don’t meet both of those criteria send the patient to state *A*, and the fraction of those remaining courses that contain both a disruptive and an inhibitory antibiotic is the parameter *σ*, which is the probability that a patient in state *A* (on antibiotics but not at elevated risk) transitions to state *V* at the conclusion of the antibiotic course (Table 1).

**Table 1:** Parameters for the antibiotic and C. difficile model.

Param.	Description	Value	Estimation method
*δ*	Rate of clearance of colonization without intervention (scaled by 6-day mean length of stay)	0.02 days^−1^	Durham et al. [15]
*β*	Baseline acquisition of colonization rate (scaled by 6-day mean length of stay)	0.1023	Calibrated to data § 2.3
*p*	Baseline rate of progression from colonized to infected (scaled by 6-day mean length of stay)	0.0353	Calibrated to data § 2.3
*σ*	Probability that a patient on antibiotics without elevated to *C. difficile* enters the elevated risk state at the end of the antibiotic course	0.388	(See § 2.3) Magill et al.
*μ*	Rate of stopping an antibiotic course during the stay (scaled by 6-day mean length of stay)	0.3 days^−1^	Huttner et al. [33]
λ	Probability that patient on an antibiotic course has elevated to *C. difficile*	0.285	(See § 2.3) Magill et al.
*ω*	Rate of starting a new antibiotic course during the stay (scaled by 6-day mean length of stay)	0.42 days^−1^	Huttner et al. [33]
*m*_*p*_	Differential progression rate from colonized to infected for patients at increased *C. difficile* vulnerability	Low-Value: 1.29, High-Value: 3.31	Calibrated to data § 2.3
*m*_*a*_	Differential acquisition rate for patients with increased *C. difficile* vulnerability	Low-Value: 1.59, High-Value: 13.25	Calibrated to data § 2.3
*f*_*A*_	Probability of new admitted patients already on antibiotics	0.24	Rubin et al. [17]
*f*_*C*_	Probability that patients are colonized with *C. difficile* on admission	0.075	Rubin et al. [17]
*ω*_0_	Probability of starting a new antibiotic on admission	0.18	Rubin et al. [33]
*m*_*s*_	Increased shedding rate of elevated patients	1 Base Case	Toth et al. [34]
*m*_*i*_	Increased shedding of infected individuals	10 Base Case	Toth et al. [34]

### 2.2 *C. difficile* transmission dynamics model

**Fig 1:**
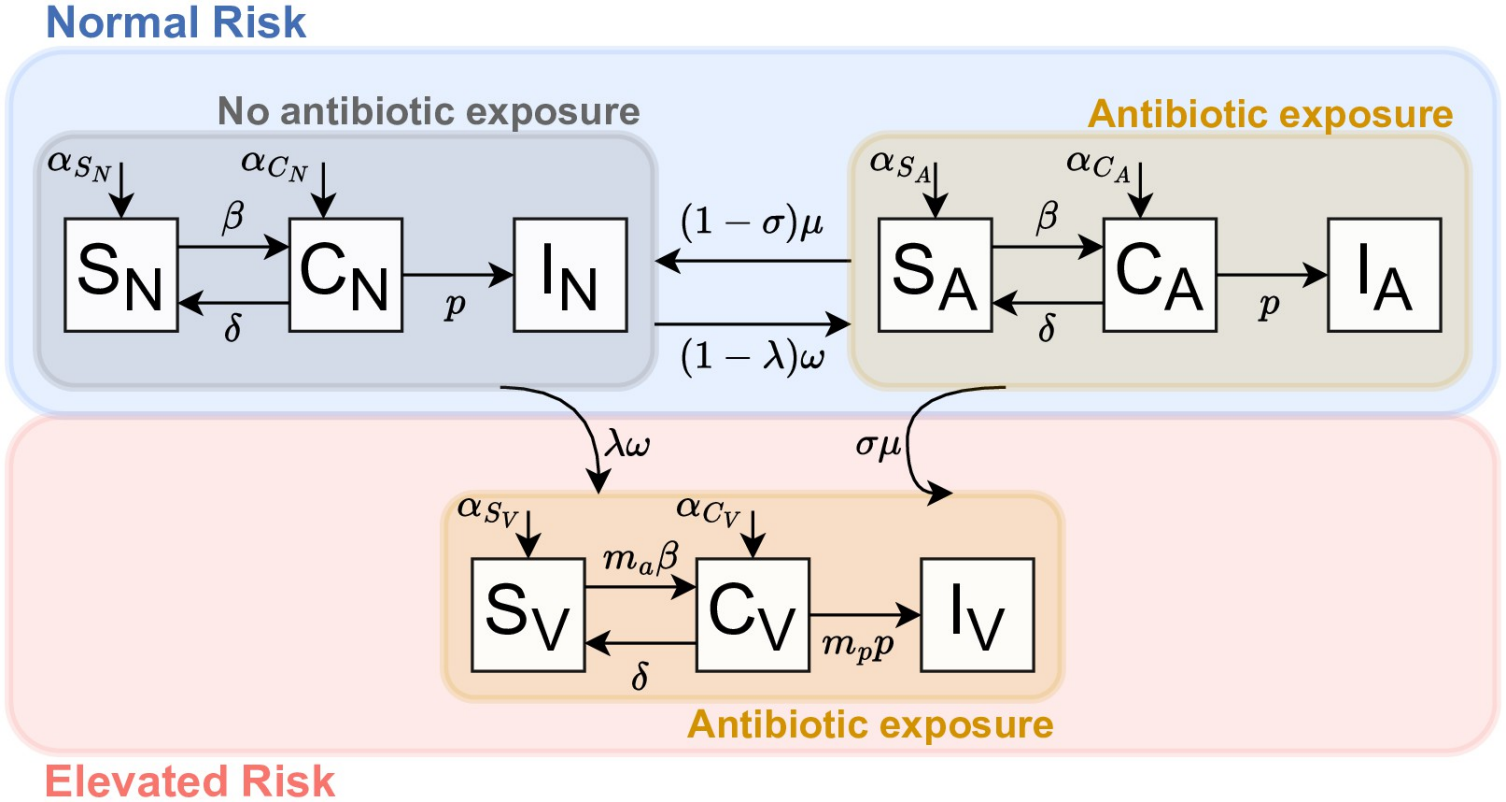
Schematic of the mechanistic model of *C. difficile* disease dynamics and antibiotic risk states. Here *αS*_*N*_ = (1 − *f*_*C*_)(1 − (*f*_*A*_ + *ω*_0_)), *αC*_*N*_ = *f*_*C*_(1 − (*f*_*A*_ + *ω*_0_)), *αS*_*A*_ = (1 − *f*_*C*_)(1 − λ)(1 − (*f*_*A*_ + *ω*_0_)), *αC*_*A*_ = *f*_*C*_(1 − λ)(1 − (*f*_*A*_ + *ω*_0_)), *αS*_*V*_ = (1 − *f*_*C*_)λ(1 − (*f*_*A*_ + *ω*_0_)), and *αC*_*V*_ = *f*_*C*_λ(1 − (*f*_*A*_ + *ω*_0_)).

We assume that different C. difficile colonization and infection dynamics prevail among individuals who are Elevated (V) and Normal (N and A). However, how individuals become elevated depends on whether they have antibiotic exposure or not (rates λ, *ω* vs *σ*, *μ* in Fig 1). This model partitions the population into “elevated” and “not elevated” based on antibiotic exposure and risk of CDI. Patients are considered to have baseline risk of *C. difficile* if they are not currently taking inhibitory antibiotics or are on a course with no disruptive or inhibitory antibiotics. Patients are considered to have increased future vulnerability but baseline current risk of *C. difficile* if they are currently taking a course of both disruptive and inhibitory antibiotics, and they are considered to have elevated if they are currently or were recently on a course with disruptive antibiotics.

Patients can transition between risk states if their antibiotic status changes. Patients can transition from either not elevated (no exposure or low-risk antibiotic exposure) to elevated by beginning an antibiotic course(at a rate λ*ω*) that includes a disruptive antibiotic or from not elevated, low-risk antibiotic risk state to elevated (at a rate *σμ*) by finishing a course of antibiotics that included disruptive and inhibitory antibiotics. Patients from not elevated, no exposure risk state can transition to not elevated, low-risk antibiotic risk state if they begin a course of antibiotics which contain an inhibitory antibiotic (at a rate (1 − λ)*ω*) and they can transition from not elevated, low-risk antibiotic risk state to not elevated no exposure risk state if they end a course of antibiotics that contain no disruptive antibiotics (at a rate (1 − *σ*)*μ*). In this paper, we assume that the gut microbiome takes longer than the duration of hospitalization to recover from CDI, and therefore once in a elevated risk state patients cannot return to not elevated [19, 32]. Further, we allow patients to be discharged from any risk state and we assume a constant discharge rate for all patients. Finally, for computational ease, we assume that the hospital population is constant (i.e., admissions equal discharges).

We model the disease dynamics of *C. difficile* patients using a *Susceptible-Colonized-Infected* compartmental model in which patients are either susceptible to *C. difficile* colonization, colonized (asymptomatic), or infected (symptomatic). Patients are further divided by their antibiotic risk states, thus we have susceptible, colonized, infected with no antibiotic exposure (*S*_*N*_, *C*_*N*_, *I*_*N*_), (Eq 2); susceptible, colonized, infected with antibiotic exposure but no increased risk of CDI (*S*_*A*_, *C*_*A*_, *I*_*A*_), (System 3); and susceptible, colonized, infected and elevated to CDI (*S*_*V*_, *C*_*V*_, *I*_*V*_), (System4). As described above, we allow individuals to transition between risk states based on starting or finishing a course of antibiotics and based on the probability that the course of antibiotics increases their vulnerability to *C. difficile*. We also assume that the system is at a steady state, so we are treating infectious classes as constant to calibrate the model. To do this we assume the equilibrium prevalence and infection incidence are constant at the steady state. This makes the model linear instead of nonlinear in order to take advantage of more mathematical techniques for solving these types of systems. The resulting *C. difficile* model is (Fig 1):

**No antibiotic exposure, not elevated**:

$$\begin{array}{c} \begin{array}{cc} \frac{dS_{N}}{dt} & =\left(1-f_{C}\right)\left(1-\left(f_{A}+\omega_{0}\right)\right)-\beta S_{N}+\delta C_{N}+\left(1-\sigma \right)\mu S_{A}-\left(\omega +1\right)S_{N}\\ \frac{dC_{N}}{dt} & =f_{C}\left(1-\left(f_{A}+\omega_{0}\right)\right)+\beta S_{N}-pC_{N}-\delta C_{N}+\left(1-\sigma \right)\mu C_{A}-\left(\omega +1\right)C_{N}\\ \frac{dI_{N}}{dt} & =pC_{N}+\left(1-\sigma \right)\mu I_{A}-\left(\omega +1\right)I_{N} \end{array} \end{array}$$ (2)

**Antibiotic exposure, not elevated**:

$$\begin{array}{c} \begin{array}{cc} \frac{dS_{A}}{dt} & =\left(1-f_{C}\right)\left(1-\lambda \right)\left(f_{A}+\omega_{0}\right)-\beta S_{A}+\delta C_{A}+\left(1-\lambda \right)\omega S_{N}-\left(\mu +1\right)S_{A}\\ \frac{dC_{A}}{dt} & =f_{C}\left(1-\lambda \right)\left(f_{A}+\omega_{0}\right)+\beta S_{A}-pC_{A}-\delta C_{A}+\left(1-\lambda \right)\omega C_{N}-\left(\mu +1\right)C_{A}\\ \frac{dI_{A}}{dt} & =pC_{A}+\left(1-\lambda \right)\omega I_{N}-\left(\mu +1\right)I_{A} \end{array} \end{array}$$ (3)

**Antibiotic exposure, elevated**:

$$\begin{array}{c} \begin{array}{cc} \frac{dS_{V}}{dt} & =\left(1-f_{C}\right)\lambda \left(f_{A}+\omega_{0}\right)-m_{a}\beta S_{V}+\delta C_{V}+\lambda \omega S_{N}+\sigma \mu S_{A}-S_{V}\\ \frac{dC_{V}}{dt} & =f_{C}\lambda \left(f_{A}+\omega_{0}\right)+m_{a}\beta S_{V}-m_{p}pC_{V}-\delta C_{V}+\lambda \omega C_{N}+\sigma \mu C_{A}-C_{V}\\ \frac{dI_{V}}{dt} & =m_{p}pC_{V}+\lambda \omega I_{N}+\sigma \mu I_{A}-I_{V} \end{array} \end{array}$$ (4)

For individuals who are not considered elevated to CDI, patients become colonized at a rate *β*, clear colonization at a rate *δ*, and progress from colonization to symptomatic infection at a rate *p*. For individuals who are at elevated to CDI, patients become colonized at a rate *βm*_*a*_, where *m*_*a*_ is the differential acquisition rates for patients on antibiotics that contain disruptive and no inhibitory antibiotics or recently completed an antibiotic course that contains both disruptive and inhibitory antibiotics; patients clear colonization at the same rate *δ*; and they progress from colonization to symptomatic infection at a rate *pm*_*p*_, where *m*_*p*_ is the differential progression rate from colonized to infected for patients at increased risk. A list of all of the states and parameter definitions, values, and whether they were estimated from data or derived from literature values can be found in Table 1. All code for the model and figures created for this paper can be found at https://github.com/Keeganlt/ABX_effects.

### 2.3 Model parametrization and calibration

We parameterize our model through a combination of extracting literature values and through simulation with calibration targets.

For the antibiotic risk state model, there are four parameters governing patients starting and stopping an antibiotic course of any type. To fix those values we use data from Veterans Affairs Hospitals [17, 33]. These data showed that 24% of admitted patients were already on antibiotics at hospital admission (*f*_*A*_ = 0.24). An additional 18% of admitted patients started a new course of antibiotics upon admission (*ω*_0_ = 0.18). The patients who were not already on or starting antibiotics at admission were prescribed a new course of antibiotics at a rate of 0.07 per day (*ω*), and inpatients finished a course of antibiotics at a rate of 0.05 per day (*μ*).

The antibiotic risk state model also requires values for two parameter governing the fraction of patients who enter a state of elevated risk for C. difficile at the beginning or end of their antibiotic course in the hospital. Those values are determined by the fraction of antibiotic courses that contain one or more drugs in the disruptive and inhibitory categories, as described in Section 2.1. McGill et al. [35] used one-day prevalence studies to determine the antimicrobial use prevalence in US hospitals in 2011. We used data from this study to derive assumptions for the frequency the antibiotic courses consisting of drugs in the above categories. Of all antibiotic drugs that in-patients were receiving on the day of a U.S. national point-prevalence survey, 15.2% were fluoroquinolones and 13.4% were 3rd/4th generation cephalosporins (disruptive);11.8% were penicillin combinations (both disruptive and inhibitory); and 6.5% were metronidazole, 3.4% were carbapenems, 2.6% were penicillins, and 1.3% were oral vancomycin (inhibitory). This survey also recorded the frequency with which patients were on different numbers of distinct antibiotics at the same time. We applied these numbers to a simulation in which we generated random antibiotic courses consisting of one or more drug categories, and we tallied a fraction of random courses that fell into the following categories:

- Containing disruptive antibiotics and no inhibitory antibiotics (28.5%)
- Containing both disruptive and inhibitory antibiotics (27.7%)
- Containing no disruptive antibiotics (43.8%)

The 28.5% of courses containing disruptive but no inhibitory antibiotics are assumed to send the patient into an elevated C. difficile risk state at the beginning of the course. Therefore, we assume that λ = 0.285 is the fraction of patients starting a hospital stay on antibiotics or starting a new course of antibiotics during the stay who enter state *V*. The remaining 1 − λ = 0.715 fraction of patients starting inpatient antibiotics enter state *A* (on antibiotics but not currently at elevated risk). Among the patients in state *A*, a portion (27.7% / 71.5% = 38.8%) are taking an antibiotic in the disruptive group but are not at elevated risk because they are also taking an inhibitory antibiotic that is assumed to cancel the disruptive effect until the antibiotic course is ended. Thus, the fraction *σ* = 0.388 of patients from state *A* enter the elevated risk state *V* upon completion of their course of antibiotics, the fraction 1 − *σ* = 0.612 enter to the normal risk state *N*.

To parameterize the SCI model, we assume that 7.5% of incoming patients are colonized (*f*_*C*_), the remaining are susceptible, and the fraction of patients entering the hospital in an infected state is negligible [17]. We assume a constant discharge rate of 0.16667/day (*γ*) [19]. Patients clear colonization without intervention at a rate of 0.02/day (*δ*) [11].

After extracting the parameters above from literature-reported values, we are left with four unknown parameters: the baseline acquisition *β*, progression to infection *p*, and the differential multipliers for acquisition *m*_*a*_ and progression *m*_*p*_ among individuals in state *V* (elevated risk). The *m*_*a*_ and *m*_*p*_ parameters quantify the increase in acquisition and progression as a result antibiotic-induced disruption. We are most strongly interested in the relationship between *β*, *p*, *m*_*a*_ and *m*_*p*_.

The disease model is calibrated using data from Fridkin et al. [11] based on the risk ratio for inpatient CDI onset between patients who had been exposed to high-risk antibiotics and patients with no recent antibiotic exposure of any kind. That study found that patients in the former category were 3.6 times more likely to progress to CDI, after controlling for time of hospital stay and other factors. For an inpatient who has not yet had infection onset at time *t* into the stay, the probability of infection onset in a short time interval is equal to the probability that the patient is colonized at time *t* times the probability of infection onset in the interval. We calculated this value separately for patients who are in the elevated risk state and patients who had no recent antibiotic exposure at all.

We focus on the relationship between the multipliers, *m*_*p*_ and *m*_*a*_. We set out to solve the system, at steady state, in terms of *p*, *β*, *m*_*p*_, and *m*_*a*_, (See Table 1) assuming all the other parameters are fixed. We introduce three constraints to use when calibrating.

The first two constraints are the equilibrium prevalence (*Y*, the fraction of the hospital population at equilibrium who are either colonized or infected) and the equilibrium infection incidence (*X*, the rate of new infection onsets occurring among patients in the hospital). These aggregate levels of colonization and infection are relatively easy to extract from data, where we obtain through observation the values *Y* = 0.16 [17] and *X* = 14.5 × 10^−4^ patient days [17]. Then the equilibrium prevalence *Y* and equilibrium infection incidence *X* can be expressed in terms of the model parameters and equilibrium variable values as in Eq 5.

$$\begin{array}{c} \begin{array}{cc} Y & =C_{N}^{⋆}+C_{A}^{⋆}+C_{V}^{⋆}+I_{N}^{⋆}+I_{A}^{⋆}+I_{V}^{⋆}\\ X & =p(C_{N}^{⋆}+C_{A}^{⋆}+m_{p}C_{V}^{⋆}). \end{array} \end{array}$$ (5)

Given the two constraints forcing *Y* and *X* to be constant, if we assume values for two of the unknown parameters, the remaining two unknowns can be determined. It is cleanest to express the relationship between the four parameters (as well as the equilibrium state variable values), by assuming the values of *p* (the baseline rate of progression from colonized to infected), and *β* (the baseline acquisition rate) are known and solving for *m*_*a*_ and *m*_*p*_. Because the equations involving the non-elevated-risk states (*N* and *A*) do not depend on the others, we can explicitly solve for the equilibrium.

We derived the formulas for each probability used to calculate the risk ratio using Mathematica [36]. To arrive at a single calibration target value representing the risk ratio, we average the time-of-stay-dependent risk ratio *R*(*t*) by integrating the product of *R*(*t*) and the fraction of patients who are still in the hospital who have not yet experienced infection onset at time *t* after admission, we call the result of this integration $\bar{R}$.

The constraint involving the risk ratio for infection onset is expressed by comparing the risk of infection onset for patients who are not yet infected and a) in an elevated risk state due to current or previous antibiotic exposure (*V*) vs. b) have no current or recent antibiotic exposure of any kind (*N*). We assume that the former group of patients is represented by those not yet infected and in state *V*, while the latter group consists of a subset of those in state *N*, i.e. it includes only those patients who entered the hospital in state *N* and never left that state. When calculating the risk ratio due to disruptive antibiotic exposure, we constructed our calculations to avoid time-of-stay bias. Because patients with longer length of stay in the facility are both more likely to enter the antibiotic-induced elevated state and more likely to progress to CDI due to longer time at risk, we must factor out the time of stay as a common factor contributing to CDI risk. Thus, we require formulas for the following probabilities as functions of time t into a patient’s stay, given that discharge has not yet occurred.

1.
$P_{S_{V}}\left(t\right)$ : probability of being in the elevated risk state and susceptible at time *t*.
2.
$P_{C_{V}}\left(t\right)$ : probability of being in the elevated risk state and colonized at time *t*.
3.
$P_{S_{\bar{N}}}\left(t\right)$ : probability of never leaving state *N* from time 0 to *t*, and being susceptible at time *t*.
4.
$P_{C_{\bar{N}}}\left(t\right)$ : probability of never leaving state *N* from time 0 to *t*, and being colonized at time *t*.

We represent the group of patients who are not in the elevated risk state and have never been exposed to antibiotics during their stay (*S*_*N*_, *C*_*N*_, and *I*_*N*_) collectively as $\bar{N}$ (Fig 1).

Therefore, the rate of observing non-infected patients experience infection onset while being in state *V* or $\bar{N}$ at stay-time *t* is the product of the probability of being colonized (because susceptible patients cannot progress directly to infection) at stay-time *t* and the progression rate from colonized to infected. Thus, the risk ratio at stay-time *t* is expressed

$$\begin{array}{c} R\left(t\right)=m_{p}\frac{P_{C_{V}}\left(t\right)/(P_{S_{V}}\left(t\right)+P_{C_{V}}\left(t\right)}{P_{C_{\bar{N}}}\left(t\right)/\left(P_{S_{\bar{N}}}\left(t\right)+P_{C_{\bar{N}}}\left(t\right)\right)} \end{array}$$ (6)

To calculate the overall risk ratio, $\bar{R}$ across all possible times of patient stays, we average the time-of-stay-dependent risk ratio *R*(*t*) by integrating the product of *R*(*t*) and the fraction of patients who are still in the hospital, have not yet experienced infection onset, and are in state $\bar{N}$ or *V*, at time *t* after admission (see S1 File for equation).

We represent the non-linear effects of these changes on altering patient-to-patient transmission by $\hat{\beta }$, where $\hat{\beta }=\frac{\beta }{Y}$ (where *Y* = (*C*_*N*_ + *C*_*A*_ + *C*_*V*_ + *m*_*I*_(*I*_*N*_ + *I*_*A*_ + *I*_*V*_) is the target prevalence from the calibration). Based on our previous work [34], we assume that infected individuals are 10 times more infectious than colonized patients (*m*_*I*_ = 10). With the intervention-altered antibiotic prescribing parameters, we allow $\beta =\hat{\beta }(C_{N}+C_{A}+C_{V}+m_{I}\left(I_{N}+I_{A}+I_{V}\right)$ to reach equilibrium, and calculate the new equilibrium colonization prevalence and CDI incidence.

After fixing one of the values of *p*, *β*, *m*_*p*_, and *m*_*a*_, we use the three constraints to calculate the value of the others necessary to meet the targets they describe. This gives us a curve in the *m*_*p*_, *m*_*a*_ plane representing all the pairs of assumptions that can satisfy the three constraints. For the following analysis, we assume that the system is at the equilibrium ($S_{N}^{⋆},\;S_{A}^{⋆},\;S_{V}^{⋆},\;C_{N}^{⋆},\;C_{A}^{⋆},\;C_{V}^{⋆}$, $I_{N}^{⋆},\;I_{A}^{⋆},\;I_{V}^{⋆}$). Using the parameter values in Table 1.

### 2.4 Alternative parameratization

In the initial model construction, we assume infected individuals are 10 times more infectious than colonized individuals. We chose this based on previous modeling results [34], however, we also consider alternatives where infected individuals are equally infectious as colonized individuals and where antibiotic risk state impacts colonization.

To do this, we consider three alternative cases.

- We assume that infected individuals are equally infectious as colonized patients (*m*_*I*_ = 1), such that $\beta =\hat{\beta }\left(C_{N}+C_{A}+C_{V}+I_{N}+I_{A}+I_{V}\right)$
- Patients in an antibiotic-induced elevated risk state are 3 times more infectious than other infected or colonized patients (*m*_*S*_ = 3), such that $\beta =\hat{\beta }(C_{N}+C_{A}+m_{S}C_{V}+m_{I}\left(I_{N}+I_{A}+m_{S}I_{V}\right)$.
- We look at the combined effect of cases 1 and 2, such that infected individuals equally infectious as colonized patients (*m*_*I*_ = 1) and antibiotic-induced elevated risk state individuals are 3 times more infectious than not elevated patients are (*m*_*S*_ = 3), such that $\beta =\hat{\beta }\left(C_{N}+C_{A}+m_{S}C_{V}+I_{N}+I_{A}+m_{S}I_{V}\right)$

### 2.5 Intervention

We are interested in both determining the relationship between antibiotic exposure and the acquisition or progression to CDI and in exploring potential interventions related to antibiotic stewardship. To do this, we have constructed three intervention strategies. First, we explore reducing the overall antibiotic prescribing rate by up to half, then we explore reducing the duration patients are on high-risk antibiotics by up to half, and finally, we explore reducing the rate at which high-risk antibiotics are prescribed by up to half.

## 3 Results

**Fig 2:**
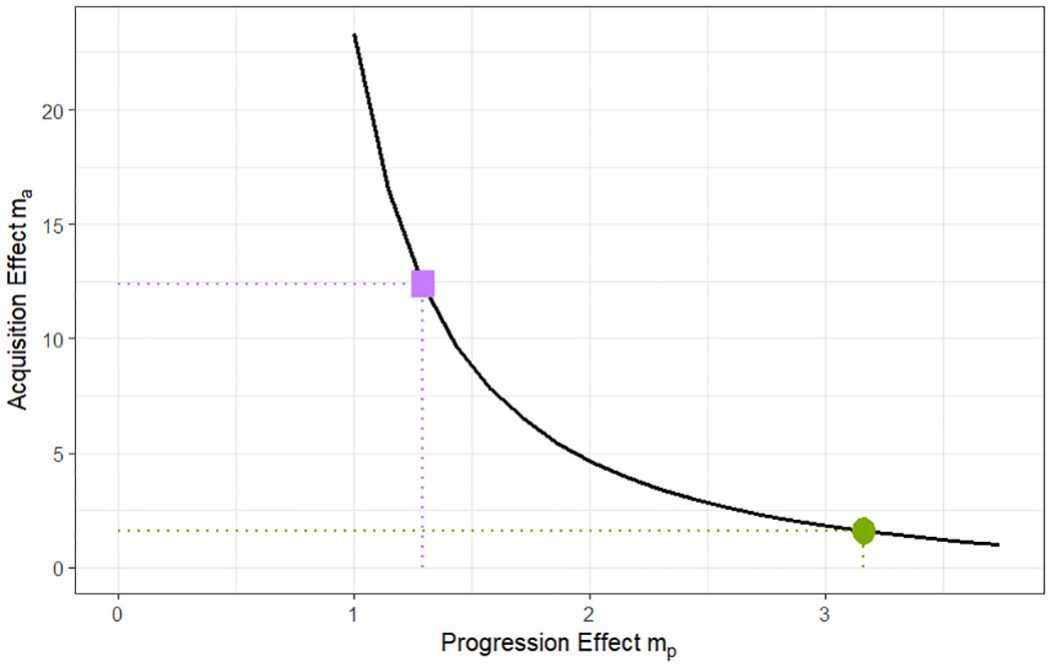
Plot of the possible values of the differential progression rate and the differential acquisition rate that satisfy all of the constraints. The relationship between the differential progression rate from colonization to infection for patients with elevated, *m*_*p*_, and the differential acquisition rate for patients with elevated, *m*_*a*_ for values of *β* and *p* calibrated to the given targets. The curve represents all possible combinations of *m*_*a*_ and *m*_*p*_ that satisfy the calibration targets. For our analysis we select two points along this curve, represented by the solid purple square (*m*_*a*_ = 13.25 and *m*_*p*_ = 1.29) and solid green circle (*m*_*a*_ = 1.59 and *m*_*p*_ = 3.31), and extract their associated values of *m*_*a*_ and *m*_*p*_ to estimate the impact of interventions. The purple square represents a higher acquisition effect and the green circle represents a higher rate of progression to CDI compared to acquisition as can be seen from their numerical values above.

We solve this problem described in Section 2.3 to obtain a relationship between *m*_*a*_ and *m*_*p*_ and find that *m*_*a*_ and *m*_*p*_ are not identifiable under the current constraints. Our results are a curve representing all of the pairs of assumptions about *m*_*a*_ and *m*_*p*_ that can satisfy the three constraints (Fig 2). Each point on the curve represents a value for *m*_*a*_ and *m*_*p*_ that satisfies the target risk ratio. For the remainder of our analysis to study the effects of certain interventions, we selected two points, one representing a higher effect on acquisition (Fig 2 purple square) and the other representing higher effect on progression (Fig 2 green circle). We use the values of *m*_*a*_ and *m*_*p*_ associated with those two points on the curve for all further analysis.

### 3.1 Antibiotic stewardship

**Fig 3:**
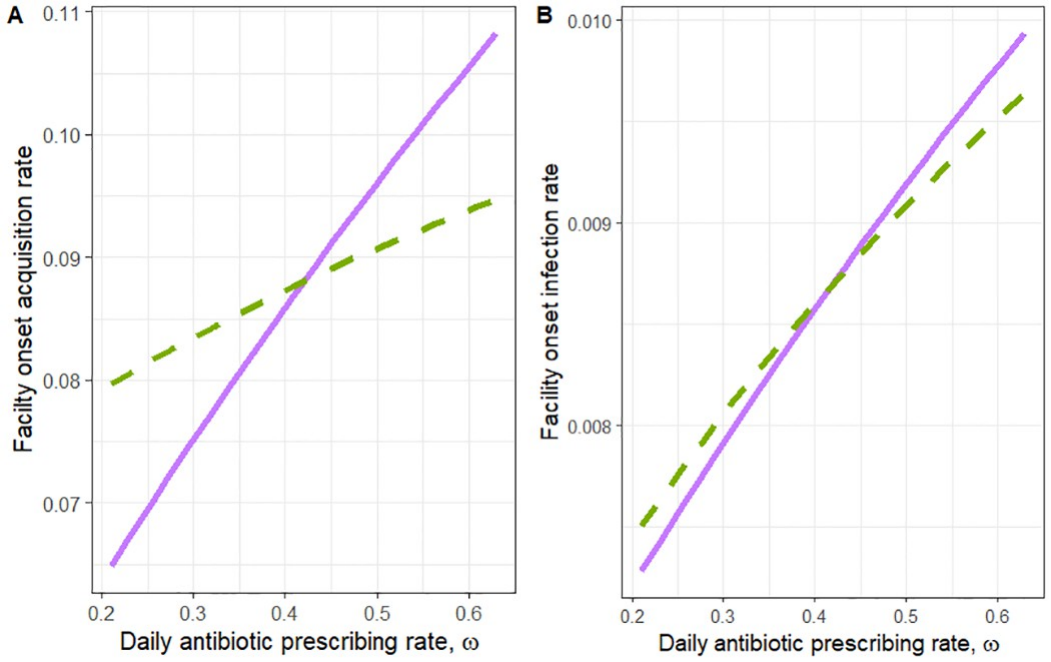
Plot of the intervention of reducing the overall prescribing rate. The relationship between the overall antibiotic prescribing rate (*ω*) and the (a) rate of facility-onset acquisition and (b) rate of facility-onset infection. The solid line depicts the relationship between antibiotic prescribing (assuming higher vulnerability to acquisition) and either facility-onset acquisition or infection when using the values of *m*_*a*_ and *m*_*p*_ pulled from the purple square (*m*_*a*_ = 13.25 and *m*_*p*_ = 1.29) on the curve in Fig 2 and the dotted line corresponds to the same relationship (assuming higher vulnerability to progression) as the solid line but for the values of *m*_*a*_ and *m*_*p*_ pulled from the green circle (*m*_*a*_ = 1.59 and *m*_*p*_ = 3.31) on the curve in Fig 2.

We find that the impact of two antibiotic stewardship interventions on facility acquisition and progression from colonization is as expected, by decreasing the overall antibiotic prescribing rate both the rate of acquisition and the rate of progression to CDI decrease (Fig 3). While we find that both the acquisition rate and the rate of progression to CDI decrease with decreased antibiotic prescribing, we find our results are not robust to the assumptions on the relationship between *m*_*p*_ and *m*_*a*_, the scaling factors for increased progression and increased acquisition for patients in the elevated risk state (Fig 3). While all choices of (*m*_*a*_, *m*_*p*_) pairs on the curve depicted in Fig 3 produce the same overall CDI risk ratio for antibiotic exposure, the predicted effect of reducing antibiotics is sensitive to which point is chosen. If it is assumed that the increased risk of being in the elevated state is more attributable to increased risk of acquisition than to increased risk of progression to CDI (*m*_*a*_ is larger than *m*_*p*_), then decreasing antibiotic prescribing has a much stronger effect on both facility-onset infection and facility-onset colonization compared to the reverse assumption.

**Fig 4:**
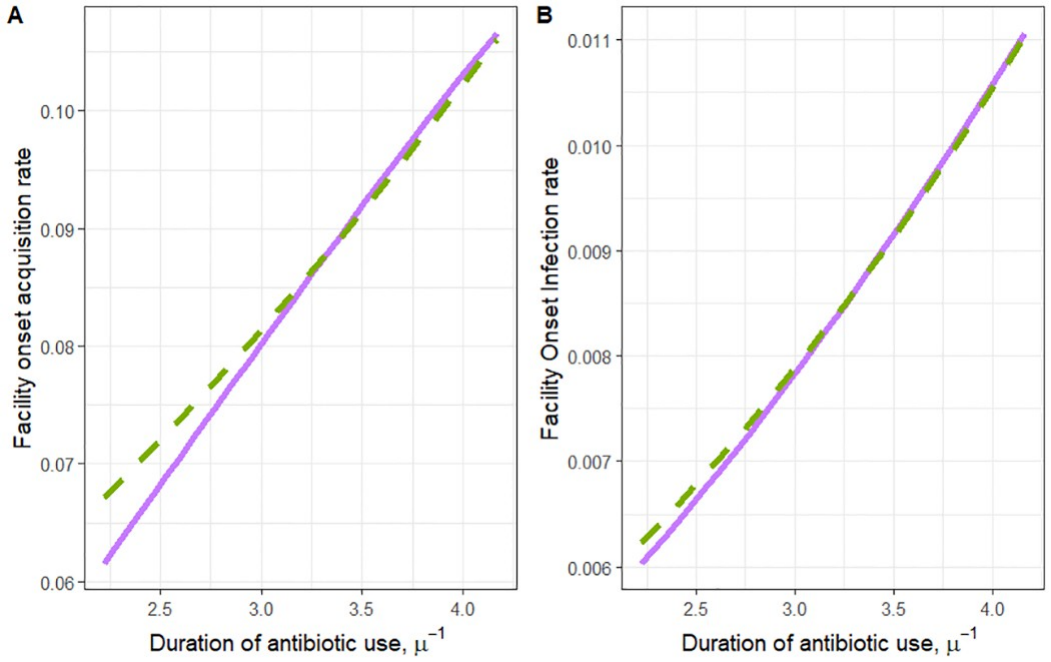
Plot of the intervention of shortening the duration of antibiotic courses. The relationship between the duration patients are on a course of antibiotics (*μ*) and the (a) rate of facility-onset acquisition and (b) rate of facility-onset infection. The solid line depicts the relationship between antibiotic prescribing (assuming higher vulnerability to acquisition) and either facility-onset acquisition or infection when using the values of *m*_*a*_ and *m*_*p*_ pulled from the purple square (*m*_*a*_ = 13.25 and *m*_*p*_ = 1.29) on the curve in Fig 2 and the dotted line corresponds to the same relationship (assuming higher vulnerability to progression) as the solid line but for the values of *m*_*a*_ and *m*_*p*_ pulled from the green circle (*m*_*a*_ = 1.59 and *m*_*p*_ = 3.31) on the curve in Fig 2.

The other intervention we explored was the impact of shortening the duration of high-risk antibiotics. What we find is counter-intuitive: by shortening the duration individuals are on high-risk antibiotics we find that the facility-onset acquisition rate and the facility-onset progression are both increased (Fig 4). Similar to intervening on the rate of antibiotic prescribing, the assumptions about the relationship between *m*_*p*_ and *m*_*a*_, the scaling factors for increased progression and increased acquisition for patients in the elevated risk state dictate the strength of the intervention. By assuming that *m*_*a*_ is larger than *m*_*p*_ shortening the duration of high-risk antibiotics increases the rate of facility-onset acquisition and the facility-onset infection rate compared to the assumption that *m*_*p*_ is larger than *m*_*a*_.

Finally, we explored the impact of shifting antibiotic prescriptions from high-risk antibiotic classes to low-risk antibiotic classes. As expected, shifting from high-risk to low-risk antibiotics decreases both the rate of facility-onset infections and colonizations (Fig 4). The assumptions on the relationship between *m*_*p*_ and *m*_*a*_ still impact the predicted effectiveness of the intervention, and by assuming that *m*_*a*_ is larger than *m*_*p*_ the effectiveness of the intervention is estimated to be larger than by assuming *m*_*p*_ is larger than *m*_*a*_.

### 3.2 Alternative parameratization

For each of the alternative cases, we explore the impact of reducing the antibiotic prescribing rate by 25%. We then calculate the equilibrium values for each of these cases and determine the change in facility acquisitions and facility-onset infections.

We quantify the impact of reducing the antibiotic rate by 25% for each alternative model case and for both assumptions about *m*_*a*_ and *m*_*p*_. Our findings are consistent with intuition: reducing antibiotic prescribing had a similar impact on the number of facility-onset colonizations and infections with increased transmissibility from antibiotics and infections with only increased transmissibility from antibiotics. Further, the assumptions about the relationship between *m*_*a*_ and *m*_*p*_ biased the results. When *m*_*p*_ is larger than *m*_*a*_, the impact of stewardship intervention is smaller than when *m*_*a*_ is assumed to be greater than *m*_*p*_.

## 4 Discussion

While mathematical models have the potential to provide important insight into the spread and control of infectious diseases, the assumptions made when employing these models must be well understood. This is an example of how subtle changes in the assumptions and resulting parameter estimates drive what appear to be contradictory results. We show that the conflicting results in the previous literature [9, 14–20] (see S3 Table in S1 File) may be entirely attributable to the assumptions about the mechanisms underlying antibiotic effects.

The main finding of our paper is that when calibrating our model to given targets including the equilibrium disease prevalence, equilibrium infection prevalence, and the risk ratio, we find that the two key parameters, the differential rate of progression (*m*_*p*_) and the differential rate of acquisition (*m*_*a*_) of elevated risk individuals are not identifiable. We find a curve that describes the relationship between these two parameters such that for every value of one parameter, there exists a value of the other parameter that satisfies the constraints imposed (Fig 2).

In this paper, we show that this identifiability problem percolates forward and leads to contradictory results for antibiotic stewardship interventions. We picked two points along that curve, one where the antibiotic effect on progression to CDI is higher (Fig 2, green circle (*m*_*a*_ = 1.59 and *m*_*p*_ = 3.31)) and one where the antibiotic effect on acquisition of colonization is higher (Fig 2, purple square (*m*_*a*_ = 13.25 and *m*_*p*_ = 1.29)) and showed that the effect of antibiotic stewardship interventions is sensitive to that choice. When we assume the effect on the acquisition of colonization is higher, interventions to reduce disruptive antibiotic usage have a higher impact.

**Fig 5:**
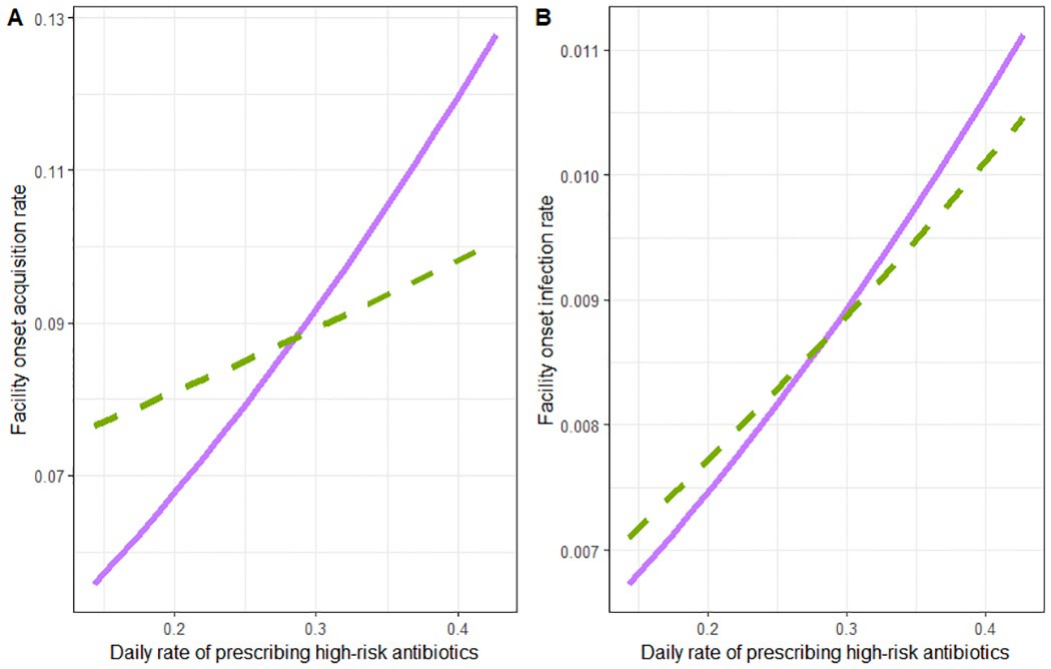
Plot of the intervention of reducing the rate of prescribing high-risk antibiotics. The relationship between the rate of prescribing high-risk antibiotics (λ) and the (a) rate of facility-onset acquisition and (b) rate of facility-onset infection. The solid line depicts the relationship between antibiotic prescribing (assuming higher vulnerability to acquisition) and either facility-onset acquisition or infection when using the values of *m*_*a*_ and *m*_*p*_ pulled from the purple square (*m*_*a*_ = 13.25 and *m*_*p*_ = 1.29) on the curve in Fig 2 and the dotted line corresponds to the same relationship (assuming higher vulnerability to progression) as the solid line but for the values of *m*_*a*_ and *m*_*p*_ pulled from the green circle (*m*_*a*_ = 1.59 and *m*_*p*_ = 3.31) on the curve in Fig 2.

We repeated this exercise for three intervention strategies: reducing the overall prescribing rate (Fig 3), shortening the duration of a course of antibiotics (Fig 4), and reducing the rate of high-risk antibiotic prescribing (Fig 5), and in each scenario we find that the results are not robust to the assumptions on the differential rate of progression and acquisition of elevated patients. We then selected three alternative model parameterizations with regard to the relative infectiousness of infected and antibiotic-elevated patients. Again, we find that the assumptions on the differential acquisition rate and the differential progression rate impacted the projected effect of antibiotic stewardship interventions (Table 2). Further, we find no predictable relationship between the assumptions made and the effectiveness of antibiotic stewardship reported by the model other than when the differential rate of progression is higher than the differential rate of acquisition, stewardship appears less effective.

**Table 2:** Reductions in facility-onset infections and facility acquisitions when the overall antibiotic prescribing rate is reduced by 25%. We compare the reduction in facility acquisitions and progressions when using the values of *m*_*a*_ and *m*_*p*_ pulled from the purple square in Fig 2 (*m*_*a*_ = 13.25 and *m*_*p*_ = 1.29) to the reduction in facility acquisitions and progressions when using the values of *m*_*a*_ and *m*_*p*_ pulled from the green circle in Fig 2 (*m*_*a*_ = 1.59 and *m*_*p*_ = 3.31). The parameters *m*_*i*_ and *m*_*s*_ are increased shedding parameters related to the cases in Section 2.4.

Outcome	Assumption effect of antibiotics	Increase from infection (Base model) *m*_*I*_ = 10 *m*_*s*_ = 1	No increase in shedding (Case i) *m*_*s*_ = *m*_*I*_ = 1	Increase from both (Case ii) *m*_*s*_ = 3 *m*_*I*_ = 10	Increase from antibiotics (Case iii) *m*_*I*_ = 1 *m*_*s*_ = 3
Reduction in acquisitions	Higher Acquisition (*m*_*a*_ = 13.25, *m*_*p*_ = 1.29)	21.5%	20.9%	31.7%	31.3%
Reduction in acquisitions	Higher Progression (*m*_*a*_ = 1.59, *m*_*p*_ = 3.31)	7.1%	3.5%	19.8%	15.3%
Reduction in infections	Higher Acquisition (*m*_*a*_ = 13.25, *m*_*p*_ = 1.29)	13.3%	12.9%	19%	18.8%
Reduction in infections	Higher Progression (*m*_*a*_ = 1.59, *m*_*p*_ = 3.31)	10%	7.7%	17.6%	14.9%

This should serve as a cautionary tale when choosing assumptions or interpreting mathematical model results. Ultimately, this highlights the importance of estimating these parameters directly from the data. Accurate measurement of the increased risk of acquiring *C. difficile* colonization and the risk of progressing to CDI while taking or upon finishing a course of antibiotics is an integral part of projecting the impact of any antibiotic stewardship interventions. Estimating these parameters should be considered a high priority.

In addition to highlighting the impact of assumptions on the effect of antibiotic stewardship efforts, our model also showed that by reducing the duration of a course of high-risk antibiotics, the facility-onset acquisition and progression rates both increase. This result, although counter-intuitive, shows that by reducing the duration of antibiotics, more patients are shifting to the elevated state while still being admitted to the hospital rather than moving to the elevated state upon or after discharge. While we did not consider vancomycin (used to treat CDI) as having a disruptive effect on gut microbiota, some results have shown a potential elevated post-treatment risk [22–26]. Thus, a similar insight might apply to vancomycin-treated CDI patients who spend additional time in the hospital after treatment, when they might be at elevated risk for reacquiring C. difficile and/or progressing to CDI. Thus, while this result shows an increase in *facility-onset* cases, it does not imply an increase in total cases. While it may appear as if this stewardship effort is negatively impacting *C. difficile* and CDI cases, it is only shifting cases to occur at the facility.

We did not consider other potential effects that disruptive or inhibitory antibiotics might have on C. difficile, such as altering the decolonization rate. Further modeling work could explore the effect of such additional factors, especially if data to support quantitative assumptions become available. Exploring these additional effects could provide a more comprehensive understanding of how antibiotics impact C. difficile dynamics and the effectiveness of stewardship interventions. Furthermore, the identifiability issues highlighted in this paper suggest that multiple parameter sets can equally explain the observed data, leading to potential variability in the projected outcomes. Our findings are based on a specific set of intervention strategies and parameter values, which may not be generalizable to all settings or populations. Future research should aim to refine these parameters through empirical data collection and validation to enhance the robustness and applicability of the model’s predictions. Finally, our study’s focus was limited to certain intervention strategies, and the results may differ under alternative strategies or in different healthcare settings. Future modeling efforts should consider a broader range of interventions and settings to provide more generalizable insights into the management of C. difficile infections.

## Supporting information

S1 File Supplemental materials. (PDF)
