# Supplementary material for: Importance of underlying mechanisms for interpreting relative risk of Clostridioides difficile infection among antibiotic-exposed patients in healthcare facilities
Source: PLoS One. 2024 Aug 8;19(8):e0306622. doi: 10.1371/journal.pone.0306622 (PMC11309424; doi:10.1371/journal.pone.0306622)
Supplement: S1 File — (PDF) [file pone.0306622.s001.pdf]

1 Supporting information

1.1 Calibration target calculation

The overall risk ratio across all possible times of patient stays is:

$$RR = \frac{\int_0^\infty RR(t)e^{-t}(P_{S_{\bar{N}}}(t) + P_{C_{\bar{N}}}(t) + P_{S_V}(t) + P_{C_V}(t))dt}{\int_0^\infty e^{-t}(P_{S_{\bar{N}}}(t) + P_{C_{\bar{N}}}(t) + P_{S_V}(t) + P_{C_V}(t))dt} \quad (1)$$

Table S1. Transition matrix for the calibration of m_a and m_p (A contains a fraction of patients recent or current on any antibiotics, $S_{\bar{N}}$, $C_{\bar{N}}$, $I_{\bar{N}}$ are defined above).

	A	$S_{\bar{N}}$	$C_{\bar{N}}$	$I_{\bar{N}}$
A	0	ω	ω	ω
$S_{\bar{N}}$	0	$-\beta - \omega$	δ	0
$C_{\bar{N}}$	0	β	$-\omega - p - \delta$	0
$I_{\bar{N}}$	0	0	p	-ω

Table S2. Transition matrix for the calibration of m_a and m_p.

	S_N	C_N	I_N	S_A	C_A	I_A	S_V	C_V	I_V
S_N	$-\beta-\omega$	δ	0	$(1-\sigma)\mu$	0	0	0	0	0
C_N	β	$-\omega-p-\delta$	0	0	$(1-\sigma)\mu$	0	0	0	0
I_N	0	p	$-\omega$	0	0	$(1-\sigma)\mu$	0	0	0
S_A	$(1-\lambda)\omega$	0	0	$-\mu-\beta$	δ	0	0	0	0
C_A	0	$(1-\lambda)\omega$	0	β	$-p-\delta-\mu$	0	0	0	0
I_A	0	0	$(1-\lambda)\omega$	0	p	$-\mu$	0	0	0
S_V	$\lambda\omega$	0	0	$\sigma\mu$	0	0	$-\beta m_a$	δ	0
C_V	0	$\lambda\omega$	0	0	$\sigma\mu$	0	βm_a	$-pm_p-\delta$	0
I_V	0	0	$\lambda\omega$	0	0	$\sigma\mu$	0	pm_p	0

1.2 Literature of Mathematical Models and Assumed Assumptions

Table S3. Mathematical models and their assumption of the antibiotic effects on acquisition and progression. Listed is the strength of the antibiotic intervention described.

**Model Citation**	**Antibiotic effect on Acquisition**	**Antibiotic effect on Progression**	**Antibiotic Intervention Effect**
Lanzas et al. [3]	Yes	Yes	N/A
Yakob et al. [2]	No	Yes	Weak
Bintz et al. [4]	Yes	Yes	Strong
Durham et al. [1]	No	Yes	N/A
Lofgren et al. [6]	Yes	No	Strong
Rubin et al. [5]	Yes	Yes	Strong
Starr et al. [7]	Yes	No	Strong
Yakob et al. [8]	No	Yes	Weak

1.3 Alternative Classification

There is evidence suggesting that the current classification of categories from the main text may need revision. Presently, we classify carbapenems as inhibitory and not disruptive. However, some evidence indicates they might actually be disruptive [9–13]. To address this, we considered a reclassification, as shown in Table 4 of the supplemental text. This table presents two scenarios: one where carbapenems are both disruptive and inhibitory, and another where they are purely disruptive without any inhibitory effect. These scenarios are illustrated in rows 2 and 3 of Table 4.

To evaluate the impact of this reclassification on interventions, we examined the first two cases from the main text, focusing on the interventions of reducing the overall prescribing rate (ω) and shortening the duration of antibiotic courses (μ). The results of this analysis are presented in Figures 1-4 of the supplemental text. Comparing these results to those in the main text, we observed minimal, if any, differences. Therefore, we conclude that while the classification is significant, it does not substantially affect the overall findings of the paper, which are primarily based on the assumed effects of antibiotics on acquisition and progression.

Table S4. Antibiotic Categories of Carbapenums

Categories	% Disruptive and No Inhibitory	% Disruptive and Inhibitory	% No Disruptive	N to V rate (λ)	A to V rate (σ)
Current Version: carbapenems are inhibitory and not disruptive	28.5%	27.7%	43.8%	0.285	0.388
Carbapenems are inhibitory and disruptive	28.6%	31.3%	40.1%	0.286	0.439
Carbapenems are disruptive and not inhibitory	33.3%	26.6%	40.1%	0.333	0.399

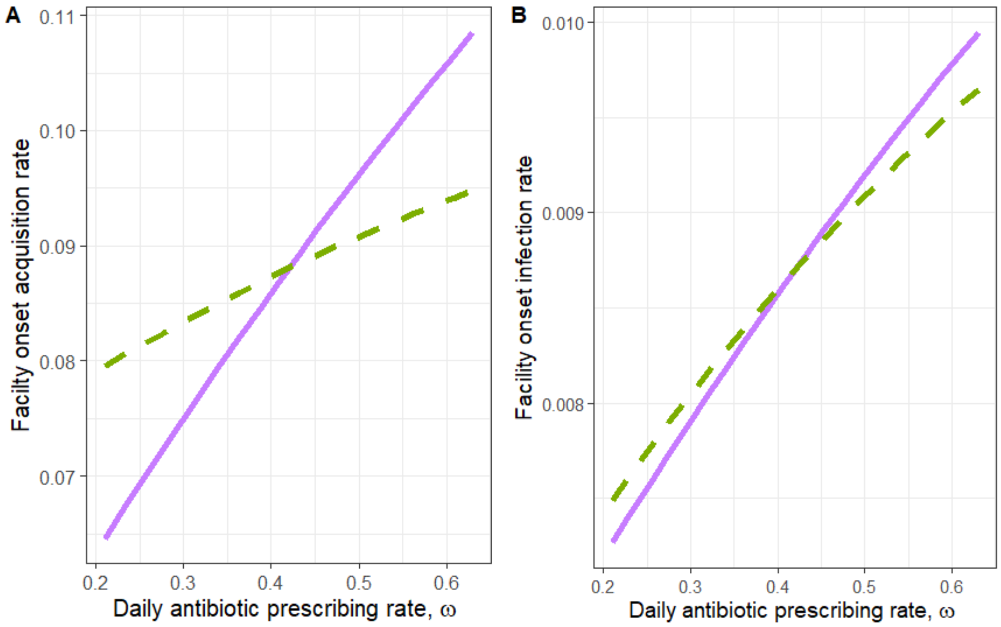

Fig S1. Plot of the intervention of reducing the overall prescribing rate using the categories from row 2 in Table S4. In this, it is considered that carbapenems are inhibitory and disruptive. The relationship between the overall antibiotic prescribing rate (ω) and the (a) rate of facility-onset acquisition and (b) rate of facility-onset infection. The solid line depicts the relationship between antibiotic prescribing (assuming higher vulnerability to acquisition) and either facility-onset acquisition or infection when using the values of m_a and m_p pulled from the purple square ($m_a = 13.25$ and $m_p = 1.29$) on the curve in Figure 2 from the main text and the dotted line corresponds to the same relationship (assuming higher vulnerability to progression) as the solid line but for the values of m_a and m_p pulled from the green circle ($m_a = 1.59$ and $m_p = 3.31$) on the curve in Figure 2 from the main text.

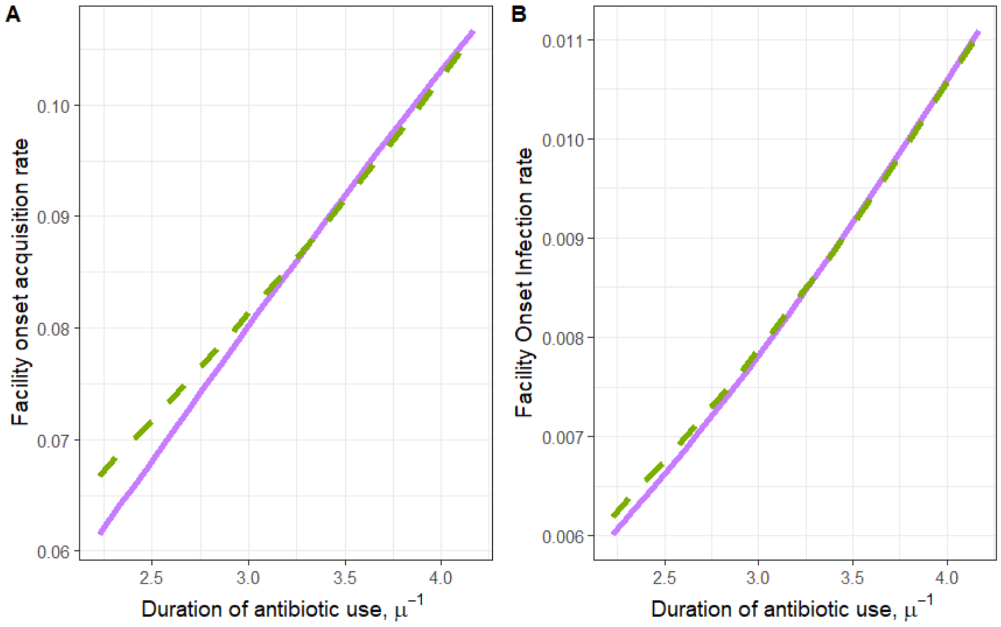

Fig S2. Plot of the intervention of shortening the duration of antibiotic courses using the categories from row 2 in Table S4. In this, it is considered that carbapenems are inhibitory and disruptive. The relationship between the duration patients are on a course of antibiotics (μ) and the (a) rate of facility-onset acquisition and (b) rate of facility-onset infection. The solid line depicts the relationship between antibiotic prescribing (assuming higher vulnerability to acquisition) and either facility-onset acquisition or infection when using the values of m_a and m_p pulled from the purple square ($m_a = 13.25$ and $m_p = 1.29$) on the curve in Figure 2 from the main text and the dotted line corresponds to the same relationship (assuming higher vulnerability to progression) as the solid line but for the values of m_a and m_p pulled from the green circle ($m_a = 1.59$ and $m_p = 3.31$) on the curve in Figure 2 from the main text.

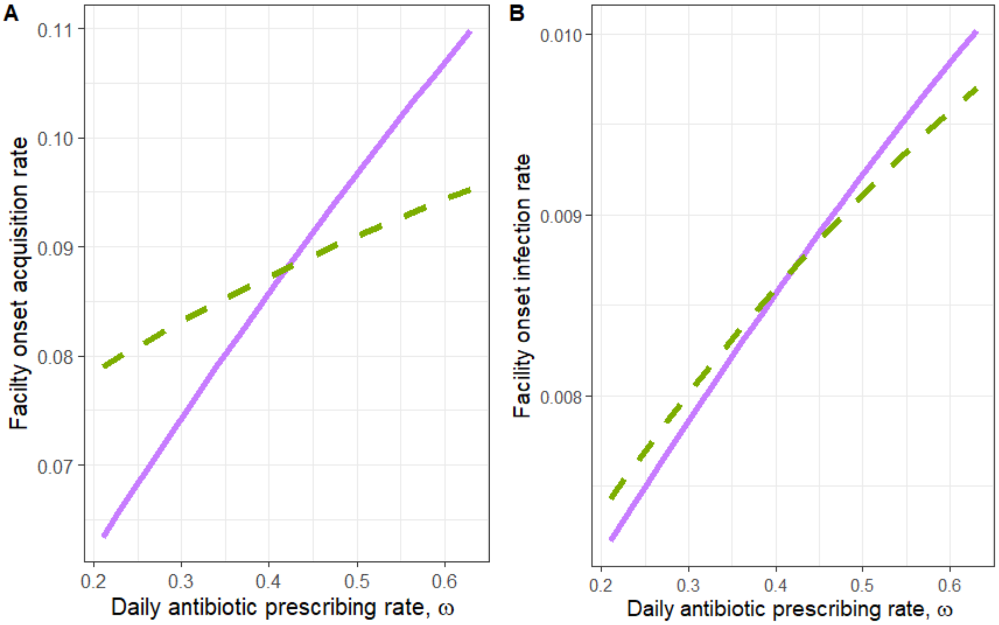

Fig S3. Plot of the intervention of reducing the overall prescribing rate using the categories from row 3 in Table S4. In this, it is considered that carbapenems are disruptive and not inhibitory. The relationship between the overall antibiotic prescribing rate (ω) and the (a) rate of facility-onset acquisition and (b) rate of facility-onset infection. The solid line depicts the relationship between antibiotic prescribing (assuming higher vulnerability to acquisition) and either facility-onset acquisition or infection when using the values of m_a and m_p pulled from the purple square ($m_a = 13.25$ and $m_p = 1.29$) on the curve in Figure 2 from the main text and the dotted line corresponds to the same relationship (assuming higher vulnerability to progression) as the solid line but for the values of m_a and m_p pulled from the green circle ($m_a = 1.59$ and $m_p = 3.31$) on the curve in Figure 2 from the main text.

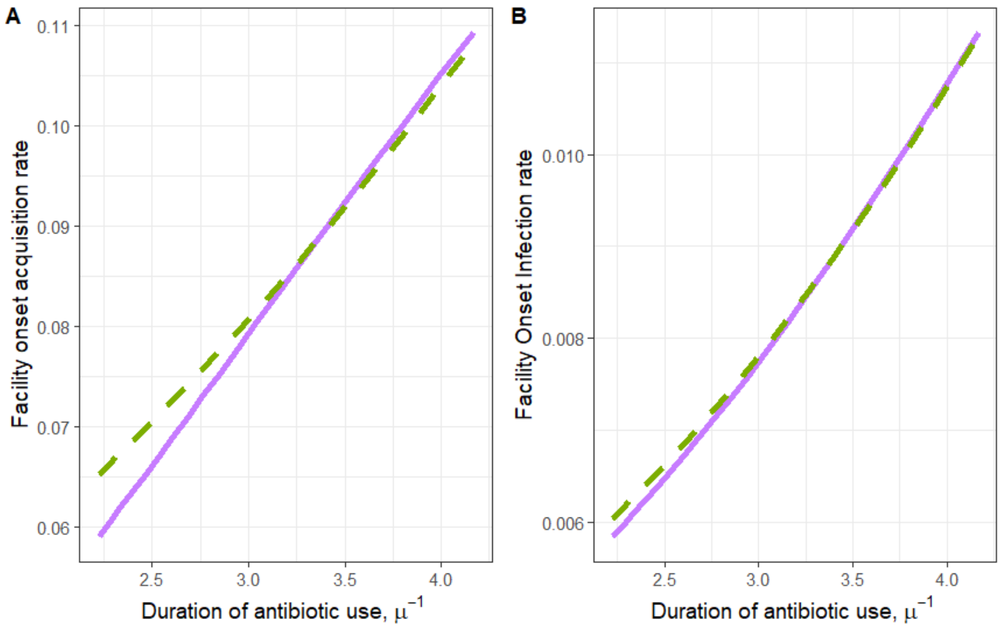

Fig S4. Plot of the intervention of shortening the duration of antibiotic courses using the categories from row 3 in Table S4. In this, it is considered that carbapenems are disruptive and not inhibitory. The relationship between the duration patients are on a course of antibiotics (μ) and the (a) rate of facility-onset acquisition and (b) rate of facility-onset infection. The solid line depicts the relationship between antibiotic prescribing (assuming higher vulnerability to acquisition) and either facility-onset acquisition or infection when using the values of m_a and m_p pulled from the purple square ($m_a = 13.25$ and $m_p = 1.29$) on the curve in Figure 2 from the main text and the dotted line corresponds to the same relationship (assuming higher vulnerability to progression) as the solid line but for the values of m_a and m_p pulled from the green circle ($m_a = 1.59$ and $m_p = 3.31$) on the curve in Figure 2 from the main text.

References

1. Durham DP, Olsen MA, Dubberke ER, Galvani AP, Townsend JP. Quantifying transmission of Clostridium difficile within and outside healthcare settings. Emerging infectious diseases. 2016; 22(4):608.

2. Yakob L, Riley TV, Paterson DL, Clements ACA. Clostridium difficile exposure as an insidious source of infection in healthcare settings: an epidemiological model. BMC infectious diseases. 2013; 13(1):376.

3. Lanzas C, Dubberke ER, Lu Z, Reske KA, Gröhn YT. Epidemiological model for Clostridium difficile transmission in healthcare settings. Infection Control & Hospital Epidemiology. 2011; 32(6):553-561.

4. Bintz J, Lenhart S, Lanzas C. Antimicrobial stewardship and environmental decontamination for the control of Clostridium difficile transmission in healthcare settings. Bulletin of mathematical biology. 2017; 79(1):36-62.

5. Rubin MA, Jones M, Leecaster M, Khader K, Ray W, Huttner A, Huttner B, Toth D, Sablay T, Borotkanics RJ, others. A simulation-based assessment of strategies to control Clostridium difficile transmission and infection. PloS One. 2013; 8(11):e80671.

6. Lofgren ET, Moehring RW, Anderson DJ, Weber DJ, Fefferman NH. A mathematical model to evaluate the routine use of fecal microbiota transplantation to prevent incident and recurrent Clostridium difficile infection. Infection Control & Hospital Epidemiology. 2014; 35(1):18-27.

7. Starr JM, Campbell A, Renshaw E, Poxton IR, Gibson, GJ. Spatio-temporal stochastic modelling of Clostridium difficile. Journal of Hospital Infection. 2009; 71(1):46-56.

8. Yakob L, Riley TV, Paterson DL, Marquess J, Clement ACA. Assessing control bundles for Clostridium difficile: a review and mathematical model. Emerging microbes & infections. 2014; 3(1):1-8.

9. Rafey A, Jahan S, Farooq U, Akhtar F, Irshad M, Nizamuddin S, Parveen A. Antibiotics Associated With Clostridium difficile Infection. Cureus. 20123; 15(5).

10. Ballo O, Kreisel EM, Eladly F, Brunnberg U, Stratmann J, Hunyady P, Hogardt M, Wichelhaus TA, Kempf VAJ, Steffen B, Vehreschild JJ, Vehreschild MJGT, Finkelmeier F, Serve H, Brandts CH. Use of carbapenems and glycopeptides increases risk for Clostridioides difficile infections in acute myeloid leukemia patients undergoing intensive induction chemotherapy. Ann Hematol. 2020; 99(11):2547-2553.

11. Vardakas KZ, Trigkidis KK, Boukouvala E, Falagas ME. Clostridium difficile infection following systemic antibiotic administration in randomised controlled trials: a systematic review and meta-analysis. Int J Antimicrob Agents. 2016; 48(1):1-10.

12. Stevens V, Dumyati G, Fine LS, Fisher SG, van Wijngaarden E. Cumulative antibiotic exposures over time and the risk of Clostridium difficile infection. Int J Antimicrob Agent. 2011; 53(1):42-48.

13. Yingchao C, Danfeng D, Lihua Z, Daosheng W, Cen J, Qi N, Chen W, Enqiang M, Yibing P. Risk factors for Clostridioides difficile infection and colonization among patients admitted to an intensive care unit in Shanghai, China. BMC Infectious Diseases. 2019; 19(1):961.
